# The Lyme Disease Vaccine Paradox

**DOI:** 10.3390/jcm15041634

**Published:** 2026-02-21

**Authors:** Eric L. Siegel, Stephen M. Rich

**Affiliations:** 1 Laboratory of Medical Zoology, Department of Microbiology, University of Massachusetts, Amherst, MA 01003, USA; 2 New England Center of Excellence in Vector-Borne Diseases, Amherst, MA 01003, USA; 3 MedZu, Inc., Amherst, MA 01003, USA

**Keywords:** *Borrelia* spp., *Ixodes* spp., Lyme disease vaccine, public health, tick-borne disease, ticks

## Abstract

Lyme disease causes a significant financial and health burden. Recent advances in developing new Lyme disease vaccines have renewed optimism that vaccination may serve as an additional strategy to reduce this burden. Vaccination alone will not address the broader relevant public health challenges posed by ticks. Our experience with prior Lyme disease vaccines suggests that even an effective vaccine would not fully reduce the incidence of human disease. Importantly, vaccination against *Borrelia* genospecies would not affect tick abundance or exposure risk. It would also not mitigate the transmission of other tick-borne pathogens that are proliferating in human-biting ticks. Tick-borne disease risk is shaped by the biological features of ticks and tick-borne disease agents, which differ from those of mosquitoes and mosquito-borne disease agents. Unique tick characteristics create opportunities for exposure and complicate prevention and post-exposure case management. In addition to infectious diseases, ticks are associated with non-infectious conditions such as tick paralysis and alpha-gal syndrome that will not be affected by a Lyme disease vaccine. The introduction of a Lyme disease vaccine should be viewed as one part of a broader risk management strategy. Emphasis must remain on clinical awareness and education for at-risk individuals. Personal protective behaviours, surveillance, and integrated tick control will also be essential to managing tick-associated health risks in both vaccinated and unvaccinated populations.

## 1. Introduction

Lyme disease is the most common vector-borne disease in temperate regions of the Northern Hemisphere [1]. This tick-borne disease is caused by the transmission of genetically diverse members of the *Borrelia burgdorferi* sensu lato complex [2]. Clinically distinct phenotypes are observed according to the regional distribution and tissue tropism of *Borrelia* genospecies. Lyme disease in North America is predominantly caused by *B. burgdorferi* sensu stricto (s.s.), transmitted by *Ixodes scapularis* and *I. pacificus* [3]. Early infection with *B. burgdorferi* is most often (in >70% of cases) associated with erythema migrans, a characteristic bullseye rash that presents with spirochete replication and radial dermal spread from the bite site [4]. This rash can be accompanied by non-specific symptoms (including fatigue and musculoskeletal pain) or facial palsy [5]. Late-stage Lyme disease may present with inflammatory arthritis, reflecting the arthritogenic nature of *B. burgdorferi* s.s. [6]. Disease may involve other organ systems, including cardiac and nervous systems, as spirochetes move from the bite site to immune-privileged tissues [7,8]. *Ixodes ricinus* and *I. persulcatus* are responsible for the transmission of *B. garinii, B. afzelii*, and *B. bavariensis* in Europe and Asia [9]. *Borrelia garinii* and *B. bavariensis* are more neurotropic, while *B. afzelii* is dermatotropic [10].

Most cases of Lyme disease in the early stages resolve quickly with timely antibiotic therapy. This usually consists of 10–14 days of oral doxycycline [11]. Some patients develop a post-treatment Lyme disease syndrome (PTLDS). This syndrome presents with symptoms including persistent fatigue and pain and may be non-specific [12]. Although the basis of PTLDS remains poorly understood, recent work suggests that antigen persistence may induce a post-infection inflammatory response [13]. This contrasts with the viable infection hypothesis that remains a controversial and challenging explanation for PTLDS pathogenesis [14]. The ongoing controversy around PTLDS underscores the persistent challenge in defining what constitutes Lyme disease. Regardless, the epidemiological impact of active infection and PTLDS highlights the need for evidence-based frameworks for pre- and post-exposure intervention.

When the *B. burgdorferi* spirochete was first identified in 1982, it was known that arthropods could transmit a variety of pathogens [15]. Local and global frameworks for vector-borne disease management were built around our understanding of the foremost disease agents transmitted by mosquitoes (*Plasmodium* spp., Yellow fever virus) and their dipteran relatives (*Leishmania* spp. and *Trypanosoma* spp. transmitted by sand flies and tsetse flies, respectively) [16]. Ticks were largely not considered a public health concern but were instead considered relevant for their effects on livestock health and production [17]. As Lyme disease case counts rose after its discovery in the mid-1970s, it became clear that our understanding of mosquito-borne diseases did not capture the complex tick ecology and feeding dynamics. Taking the northeastern United States as an example, *I. scapularis* maintains *B. burgdorferi* in an ecological cycle involving white-footed mice and other small mammals across the temperate zone [18]. Geographic range expanded from two localised foci decades ago (upper Midwest and northeastern United States) [19]. This expansion occurred because of significant and widespread ecological changes, such as suburban reforestation and habitat fragmentation [19]. Ecological influences enabled ticks and the vertebrate species that support their reproductive cycles, notably white-tailed deer, to expand their geographic ranges and population densities.

## 2. The Lyme Disease Vaccine

Against the ecological backdrop of tick proliferation, a half-century has passed since the first cases were described in Connecticut, United States. Lyme disease has now become the most prevalent vector-borne disease in the United States. Case counts are underreported, but estimated annual incident cases in the United States exceed 476,000 [20]. Burdens vary elsewhere. They are similarly underreported and may reach 200,000 cases per year in Western Europe alone [21]. Lyme disease has caused widespread fear in endemic areas. In many ways, this sentiment echoes a reaction observed during the polio epidemic of the mid-1900s [22]. Hope similarly mirrors the successful response to polio in that safe, effective vaccines may ease current, albeit underreported, epidemiological trends by preventing human infection with *B. burgdorferi*.

The first (and only approved to date) human Lyme disease vaccine, LYMErix, and the investigational VLA15 target *Borrelia*’s outer surface protein A (OspA) [23,24]. OspA is a surface-exposed lipoprotein essential for adhesion to the tick midgut epithelium, enabling persistence between tick blood meals [25]. Expression of *ospA* is later downregulated when *Borrelia* lives in the mammalian host. The production of antibodies targeting OspA is stimulated with these vaccines. Anti-OspA antibodies circulating in the human host will be ingested by a feeding tick and act on spirochetes in the tick lumen, preventing their migration to the salivary glands and into the human host (a transmission-blocking vaccination approach). Protection depends on sufficient circulation of anti-OspA antibodies. Immunity is therefore reliant on compliance with a full initial vaccination series and the timely administration of appropriate boosters. Peak anti-OspA levels for LYMErix and VLA15 have been demonstrated one month after the third dose of an initial series (0, 1, and 6 or 12 months for LYMErix; 0, 2, and 6 months for VLA15) [26,27]. LYMErix-induced titers decline thereafter but were maintained within protective ranges for at least one full tick season (6 months). Similar kinetics have been observed with VLA15.

The VLA15 vaccine candidate phase 3 trial is continuing [28]. Hurdles remain before vaccine implementation. Considerations relevant to the vaccine chemistry itself include durability and dosing. Strain coverage may be relevant with this vaccine, although to a lesser extent than with LYMErix. VLA15 is a hexavalent vaccine covering OspA serotypes of the most clinically relevant *B. burgdorferi* s.l. genospecies in North America and Europe, as demonstrated in preclinical models (ST1 for *B. burgdorferi* s.s. and ST2-ST6 for *B. afzelii*, *B. garinii*, and *B. bavariensis*) [24]. This is a significant improvement over LYMErix, a monovalent vaccine that narrowly targeted *B. burgdorferi* s.s. in North America. Preclinical studies indicate that higher titers may be required to block *B. afzelii* transmission [29]. The effects of this on protection will need further study. The *B. burgdorferi* s.l. complex currently includes at least 20 accepted genospecies [30]. At least eight are human-pathogenic and cause Lyme disease [30]. The number and proportion of circulating strains covered by OspA serotypes in endemic regions remains uncertain, given ongoing studies of tick and reservoir-host diversity that include several newly proposed genospecies.

Because LYMErix was available for only a few years after market introduction, and VLA15 is still investigational, long-term profiles are mostly studied in antibiotic-treated, unvaccinated patients. However, because the OspA target acts essentially as a transmission blocker, successful vaccination will prevent infection. It would also prevent host inflammatory syndromes, as seen with PTLDS, under both prevailing (infection persistence/post-treatment syndrome) [14]. Known and unknown infections at the time of vaccination would not be cleared by the OspA vaccine and could still lead to clinical disease or post-infection sequelae. These infections could progress to acute Lyme disease and require antibiotic therapy. Development of PTLDS is also possible from this, since OspA is not essential for disseminated *Borrelia* infection. In the context of possible pre-existing infections with uncertain long-term consequences or breakthrough infections resulting in seroconversion, reduced clinical vigilance or delayed evaluation in vaccinated individuals who perceive themselves to be fully protected warrants consideration.

VLA15’s effectiveness has not yet been proven in major clinical trials, though data are expected in the coming year. Even if effectiveness is demonstrated, perception and uptake are central challenges for any future Lyme disease vaccine programme. The LYMErix vaccine was 70–80% effective and was withdrawn in 2002 due to low consumer demand [31]. LYMErix was surrounded by safety concerns [32]. This was due to a theorised association with antibiotic-refractory arthritis, based on molecular mimicry between an OspA epitope and the human lymphocyte function-associated antigen-1 (LFA-1). The association was eventually disproven, but a lack of confidence in its safety and the requirement of unwanted boosters were among the reasons the vaccine was voluntarily withdrawn from the market [28]. VLA15 does not contain the epitope implicated in past LFA-1-related concerns. It is unknown how residual concerns may affect uptake with a new vaccine.

Surveys in Lyme disease endemic regions of North America consistently show that more than 60% of people are willing to receive a new Lyme disease vaccine [33]. Unwillingness in endemic regions has been associated with low confidence in general vaccination and low perceived risk/severity of Lyme disease. These trends align with the broader post-COVID climate, which has strong anti-vaccine sentiment across many demographics [34]. While awareness of vaccination has increased in the post-COVID period, longitudinal surveys have documented intensifying polarisation in attitudes toward and behaviours related to vaccination [35]. Proactively addressing concerns that may reduce uptake will need to be a priority with a new Lyme disease vaccine and others. It is unknown at this point how successful efforts may be in reversing perception trends, as surveys indicate that these individuals are unlikely to be influenced by healthcare provider recommendations or interventions [33].

Tick-borne encephalitis (TBE) vaccines may offer insight into how new Lyme disease vaccines may be received. There are multiple TBE vaccines circulating [36]. The primary vaccines used for the European subtype (TBEV-Eu) are FSME-IMMUN (as TICOVAC in the United States) and Encepur. TBE-Moscow and EnceVir target the far eastern (TBEV-FE) and Siberian (TBEV-SE) subtypes. A powerful study recently assessed awareness of and uptake of TBE vaccines, alongside vaccines for other diseases, in TBE-endemic and non-endemic European regions. This survey found consistently high awareness of TBE disease and the vaccine, but low, heterogeneous perceptions and uptake of the vaccine in endemic regions (22%, 4–69%) [37]. Compliance with recommended schedules in this study was poor. Very few (only 21%) received the full initial series, and only 7% received the first booster in endemic countries. This study supports others showing that TBE vaccination rates are much lower than those of other commonly administered vaccines, including tetanus and measles, despite similar awareness [38]. Those who reject TBE vaccination in endemic regions generally report fear of side effects and consider the vaccine unnecessary. Many also do not perceive themselves to be at risk or feel the risk is too low to warrant vaccination [39,40]. Together, these studies and others suggest that even for a tick-borne disease with effective and well-characterised vaccines, uptake and compliance are highly heterogeneous and coupled to broad confidence in vaccination. Similar variables will be present when people consider whether to vaccinate against Lyme disease, especially in the case that many of these same individuals surveyed will be targeted for Lyme disease vaccines. Messaging about vaccine safety and clarifying individual risk will be critical to mitigating the effects on Lyme disease vaccine uptake.

The impact of a new Lyme disease vaccine on compliance with personal protection recommendations will require study. A longitudinal study conducted after LYMErix became available found that vaccination was associated with a significant reduction in perceived risk [41]. Vaccinated adults reported using tick repellents and protective clothing less often than unvaccinated individuals [41]. Conversely, individuals in Switzerland and other European countries with greater awareness of and perception of TBE risk were more likely to be vaccinated and to report using other personal protection strategies [37,42]. This suggests we could see the opposite effect. Vaccination may be associated with higher adherence to recommendations due to increased awareness of the vaccination process. Depending on how the vaccine rollout interacts with risk perception, clinical messaging and communication strategies will need to emphasise the importance of maintaining compliance with guidelines.

## 3. The Fallacy of Eradication and the Persistence of Threats Associated with Ticks

The idea of reducing our risk of tick-borne diseases through vaccine development is becoming a common public belief, despite being unrealistic. This trend gestures back to the decade before Lyme disease was discovered. Eradication was the focus of efforts to address infectious disease agents at this time. Malaria was the first vector-borne disease targeted for eradication. The failure of eradication efforts and the alternative push for disease management showed that the disease agent did not need to be eradicated to greatly improve public health [43,44]. Focusing narrowly on eradication caused us to miss the opportunity to manage the enduring vector threat.

We are realistically approaching the time when a new Lyme disease vaccine will be introduced to the market. We must acknowledge the challenges that will persist and be prepared to align clinical and public health strategies accordingly, considering historical lessons that emphasise management over eradication. These considerations will center on the fact that even if Lyme disease were completely eradicated, ticks would remain abundant and widely distributed and would continue to vector multiple pathogens. An example of this is the transmission of *Babesia microti*, *Anaplasma phagocytophilum*, *Borrelia mayonii*, *Borrelia miyamotoi*, Powassan virus, and *Ehrlichia muris eauclairensis* (EME, formerly *Ehrlichia muris*-like agent (EMLA)) by *I. scapularis* [45,46,47,48]. These pathogens can cause severe disease on their own but may also be present in co-infections with *B. burgdorferi*, which can affect clinical presentations [49]. *Amblyomma americanum* is another tick species that transmits agents such as *E. chaffeensis*, *E. ewingii*, Heartland virus, and Bourbon virus [50]. Parallel dynamics in Europe and Asia further illustrate this with *I. ricinus* and *I. persulcatus* transmitting *A. phagocytophilum*, *B. miyamotoi*, TBEV, *Rickettsia* spp., and other medically relevant pathogens [51]. Collectively, these examples highlight the significance of the broader threat posed by ticks and the diverse pathogens they transmit.

## 4. Implications of Biological Nuances on Continued Tick-Borne Disease Risk Management

Human-biting tick species are proliferating and expanding their ranges, increasing the risk in previously safe areas. Public health in a world where Lyme disease vaccination is widespread would therefore still require diligence against tick-borne diseases, guided by our five decades of fight against Lyme disease. This will demand employing multiple strategies. As we proceed, we must keep in mind the fundamental biological characteristics that render ticks as persistent vectors of human disease and the implications of these traits for vector control strategies.

First, and perhaps most importantly, protection and post-exposure strategies must be designed with the understanding that tick feeding physiology differs from that of mosquitoes. Mosquitoes acquire their bloodmeal within seconds, whereas medically relevant hard ticks remain attached and feed for several days. For many tick pathogens, transmission risk directly correlates with attachment time [52]. The pathogen must cross multiple physiological barriers from the tick to the bite site, only beginning to replicate after feeding begins. Tick morphology changes occur during feeding and can provide clinically relevant indicators of attachment time. Quantitative exposure indices associated with feeding time are often useful aids for clinical decision-making [53]. It must be noted, however, that disease cases often cannot be reliably tied to self-reported recollection of bites. The preserved tick may therefore not be available to take such measures [54]. Since exposure risk increases with tick feeding time, a disease-dependent window of opportunity exists to reduce exposure. While preventing landing is a critical metric for mosquito-targeting repellents or pesticides, there is an opportunity to remove feeding ticks to improve outcomes. Therefore, as the tick threat persists in a world where Lyme disease vaccination is widespread, public health officials will need to emphasise personal protection measures, such as performing tick checks and wearing pesticide-impregnated clothing.

Second, unlike mosquitoes, ticks are long-lived and take one blood meal per life stage. In the northeastern United States, for example, *I. scapularis* has a 2-year life cycle across three life stages [54]. Mosquitoes live for only days to weeks and produce multiple generations each season. Also, unlike mosquitoes, few tick species bite humans. Some tick species show life-stage-dependent host specificity and seasonal activity. Transmission risk aligns with the phenology of the vector species responsible for transmission. Understanding seasonal parameters and discriminating between the small number of human-biting tick species can guide diagnostic testing and patient follow-up or self-monitoring. Lyme disease is also the only tick-borne disease for which prophylaxis has been shown to be effective. Doxycycline may be given orally within 72 h of a suspected exposure to prevent Lyme disease [55]. If Lyme disease vaccination is widespread, there will continue to be great variation in knowledge, attitudes, and practices/behaviours of clinicians and patients. An understanding of vector–pathogen–host interactions will remain important and require ongoing attention.

Third, for diseases with causative agents that are exclusively maintained in human hosts, such as polio and measles, high vaccination coverage interrupts transmission cycles by depriving the pathogen of susceptible hosts. Even the most effective Lyme disease vaccine cannot offer herd immunity. The enzootic maintenance of wildlife-amplified tick-borne zoonotic disease agents for which humans are dead-end hosts will remain cyclic, regardless of human vaccination. Therefore, the subclinical, vaccinated carrier of *Borrelia* is not relevant to the propagation of transmission and maintenance. This contrasts with the significance of subclinical cases in entirely anthroponotic pathogens, such as measles, or largely anthroponotic pathogens after spillover, such as COVID-19. The only operational effect of human vaccination is a reduction in reported cases, the extent of which is unknown. In the LYMErix phase 3 trial, a three-dose series induced over 70% efficacy. This protection dropped to around 50% with only two doses [56]. Reduced prospects for effectiveness were further highlighted by low uptake. Given this historical data, it is unlikely that a new vaccine alone would reduce the population-level incidence of Lyme disease by more than 50%. Messaging and intervention may serve to increase these numbers. Implementation will have to be paired with integrated management to reduce human–tick contact and suppress infected tick populations. Mitigating risk from other tick-borne pathogens will be necessary to protect the unvaccinated and vaccine non-responders. As seen with canine Lyme disease vaccines, protection will be limited to adherence to the vaccination schedule and timely boosters [57].

Fourth, in addition to their well-known historical roles as general nuisances and disease vectors, ticks are now recognised as broader human health hazards due to lesser-understood physiological and immune consequences of feeding. Not all tick-associated diseases are infectious. Tick paralysis results from exposure to salivary neurotoxins in certain species and resolves with tick removal [58]. Alpha-gal syndrome is another emerging bite-associated condition. This syndrome presents with delayed allergic reactions following red meat consumption [59]. Reactions can occur hours post-ingestion, causing gastrointestinal upset and, in some cases, may cause hives and anaphylaxis. These four considerations show that sustainable risk reduction cannot rely on vaccination alone and requires integrated strategies.

## 5. A Tick-Centric Public Health Focus

Simply put, the paradox of the Lyme disease vaccine is that success against a single pathogen will not diminish the public health consequences of ticks. Focusing on Lyme disease or framing our focus around mosquitoes will not address the full range of tick-borne threats. Vaccine deployment will undoubtedly improve the clinical and population-level landscape for Lyme disease, but the strategic posture of medicine and public health must remain tick-centric. Anti-tick vaccines are being explored as the most tick-centric vaccine strategy. These vaccines target tick-expressed epitopes, such as salivary or midgut antigens, to prevent the transmission of multiple pathogens. The Bm86-based vaccine is a successful example that targets a concealed midgut antigen of *Rhipicephalus microplus* ticks to protect cattle [60]. The delivery of anti-tick vaccines for human use is not expected in the near future.

Two recent studies have estimated the annual number of people bitten by ticks in the United States, considering a tick bite as an instance of an individual being fed on by a tick. One passive surveillance study on *I. scapularis* tick infection rates in the Northeastern United States suggested about 1.3 million people may be bitten by *Ixodes* ticks each year [61]. Another study, using nationally representative survey data, estimated the number at over 31 million [62]. The actual figure probably lies somewhere between these estimates but may never be precisely known. While widespread Lyme disease vaccination could boost morale, this hypothetical does not reflect reality, as ticks will remain abundant and pose broad health risks. Protecting vulnerable populations through personal and environmental protection measures will be especially important. These groups will include older adults, immunocompromised individuals, and those with higher exposure due to their home or work environments.

## Data Availability

No original data was generated in preparation of this manuscript.
